# Where am I in virtual reality?

**DOI:** 10.1371/journal.pone.0204358

**Published:** 2018-10-10

**Authors:** Albert H. van der Veer, Adrian J. T. Alsmith, Matthew R. Longo, Hong Yu Wong, Betty J. Mohler

**Affiliations:** 1 Max Planck Institute for Biological Cybernetics, Tübingen, Germany; 2 Graduate Training Centre of Neuroscience, University of Tübingen, Tübingen, Germany; 3 Institut Jean Nicod, DEC, ENS, EHESS, CNRS, PSL University, Paris, France; 4 Department of Psychological Sciences, Birkbeck, University of London, London, United Kingdom; 5 Werner Reichardt Centre for Integrative Neuroscience, University of Tübingen, Tübingen, Germany; 6 Philosophisches Seminar, University of Tübingen, Tübingen, Germany; 7 Institute for Sport Science, Technical University of Darmstadt, Darmstadt, Germany; 8 Max Planck Institute for Intelligent Systems, Tübingen, Germany; Anglia Ruskin University, UNITED KINGDOM

## Abstract

It is currently not well understood whether people experience themselves to be located in one or more specific part(s) of their body. Virtual reality (VR) is increasingly used as a tool to study aspects of bodily perception and self-consciousness, due to its strong experimental control and ease in manipulating multi-sensory aspects of bodily experience. To investigate where people self-locate in their body within virtual reality, we asked participants to point directly at themselves with a virtual pointer, in a VR headset. In previous work employing a physical pointer, participants mainly located themselves in the upper face and upper torso. In this study, using a VR headset, participants mainly located themselves in the upper face. In an additional body template task where participants pointed at themselves on a picture of a simple body outline, participants pointed most often to the upper torso, followed by the (upper) face. These results raise the question as to whether head-mounted virtual reality might alter where people locate themselves making them more “head-centred”.

## 1. Introduction

Generally, people locate themselves where their bodies are. Here we ask more specifically: Where do people locate themselves in their bodies? Currently it is unknown whether people locate themselves in one or more specific part(s) of their body. The specific methods used in empirical research to investigate where people locate themselves depend largely on which of several possible notions of self-location is under investigation. In the literature, at least the following dominant notions of bodily self-location can be found. (1) Self-location as the bodily location people consider to be the centre from which they perceive the world, the centre of their first-person frame of reference, or egocentre [1,2]; (2) the bodily location people experience themselves to be in relative to external space [3]; (3) the location in or on their body where people experience themselves to be, or the part(s) of their bodies people associate themselves with the most [4,5]. Whether these different conceptions of self-location can be completely distinguished experimentally, in terms of their underlying sources of information, psychological mechanisms and neuronal structures, as well as metaphysically and phenomenologically, is however a topic that requires further integration of several lines of research.

Alsmith and Longo [6] operationalised self-location as the location resulting from pointing "directly at you” in 1PP, and interpreted it as the bodily location one judges to be one’s ultimate location, which is very close to conceptualisation (3) above. Alsmith and Longo found that participants’ judgements were not spread out homogeneously across the entire body, nor is it localised in any single point. In their study, participants were asked to stop a pointer when it was pointing “directly at you”—either by manual manipulation of a physical pointer whilst blindfolded, or by visually discerning whether the physical pointer manipulated by an experimenter was in the correct position. They found two distinct regions to be judged as where "I" am inside my body: the upper face and the upper torso, according to which participants reached first.

Most literature has focused on self-location using an outline of a human body where the task does not involve pointing to oneself but rather general localization of or on another person. Limanowski and Hecht found a dominant role for the brain (which was reported most) and the heart for self-location in humans, when participants were asked to indicate the “centre of the self” by placing markers on human silhouettes [5]. Moreover, they found that most people seem to believe there is one single point inside the human body where their self is located. Starmans and Bloom, based on people's judgments of when objects are closer to a person [7] and on a task of erasing as much as possible of a picture of a stick figure named Sally, while still leaving Sally in the picture [8], argued that people locate the self mainly in the head and, more particularly, in or near the eyes. In a study using open questions and forced-choice self-localizing on a body silhouette, Anglin, on the contrary found, that some participants reported that the self is not centralized in one location and that, overall, participants tended to locate the self and mind in the head and the soul in the chest [4]. Using a more implicit method, Alsmith, Ferrè and Longo recently found evidence for the use of a weighted combination of head and torso for self-location judgments [9].

The primary aim of the current paper is to investigate where people locate themselves within their body *in virtual reality* (VR). The paradigm from Alsmith and Longo [6] was adapted with the only change being that VR technology was used. More specifically, the aim of this study was to see if the findings from Alsmith and Longo would also be found in a virtual world, particularly the two distinct locations for pointing to self, the upper torso and the upper face. To test this, we used a commonly available VR setup, a VR headset. A body template task, inspired by Limanowski and Hecht [5] and Anglin [4], was included to explore where participants point, when asked to point at themselves outside of VR and to see whether this pointing would be consistent with the self-locations found in the VR headset.

VR headsets have been increasingly used to study body ownership and body swap illusions [10–12], as well as the manipulation of specific bodily experiences [13–16]. However, the influence of VR headsets on self-location has not been thoroughly investigated and may play a role in those studies. Also, in VR environments being designed for health applications, entertainment and training/education, the user's experienced self-location may be an important factor for the intended effects to be achieved. These issues illustrate the importance of an investigation of self-location in VR.

## 2. Methods

### 2.1. Participants

Twenty-three volunteers (thirteen female; mean age: 30.0 (SD = 9.0) years, range: 20–56 years, 19 right-handed by self-report), naïve to the purpose of the experiment, participated, all with normal or corrected-to-normal vision (including stereo depth vision, tested with the Stereo fly test (Stereo Optical Co., Inc., Chicago, IL)). The participants were recruited from the participant database of the Max Planck Institute for Biological Cybernetics in Tübingen, Germany. All participants gave written informed consent. Procedures were in accordance with the principles of the Declaration of Helsinki and approved by the Ethics Committee of the University Hospital Tübingen.

**Fig 1:**
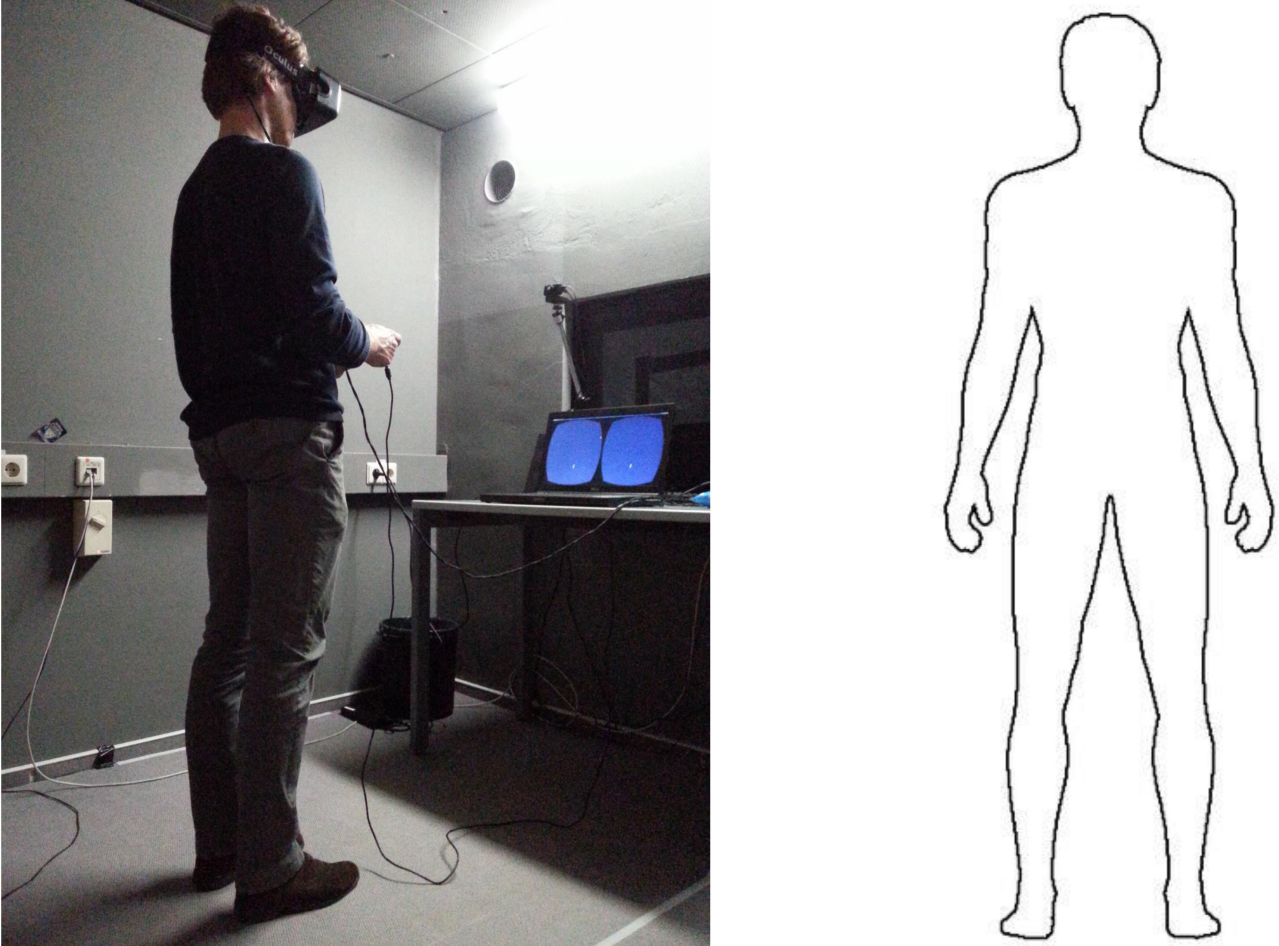
The VR headset experimental setup and the body template. (A) The participant was standing still, wearing the VR headset and holding the controller. The participant’s task was to rotate a virtual pointer in their sagittal plane until they felt it was pointing ‘directly at you’. The individual depicted has given written informed consent (as outlined in PLOS consent form) to appear identifiably in this publication (left image). (B) On this picture of an outline of a body participants were asked to "Point directly at you", under the assumption this was a picture of themselves (right image).

The individual depicted in Fig 1A has given written informed consent (as outlined in the PLOS consent form) to appear identifiably in this publication.

### 2.2. Procedure

#### VR pointing task

Participants read an information sheet and signed an informed consent form. The experimenter measured the height of the top of the participant’s head (cranial vertex), eyes (pupils), chin (gnathion), shoulders (acromion) and hips (greater trochanter), followed by a test for binocular stereo vision. The participant then put on the VR headset. After a calibration of the VR headset, the experiment began (see Fig 1A). Based on Alsmith & Longo [6] we predicted that participants would point towards the upper torso and upper face.

#### Body template task

Following the VR pointing task, a few minutes after the VR headset had been removed from them, the participants performed a body template task, where they were asked to “Point directly at you” on an A4 print of a drawn frontal body outline (see Fig 1B), under the assumption this was a picture of themselves. Based on previous literature [4,5] we predicted that participants in the body template task would point mainly towards the head and possibly also the chest.

#### Survey questions

After the two tasks, several questions about the employed strategy, demographics and psychological state were asked in a pen-and-paper survey (see S1 Post-questionnaire).

### 2.3. Experimental setup

During the experiment the participant stood in front of a table on which a Dell Precision M6700 laptop was positioned, running the experiment (see Fig 1A). The computer had an Intel Core i7-3940XM central processor running at 3.00 GHz and an NVIDIA Quadro K5000M graphics card. An Oculus Rift development kit 2 VR headset was used for stimulus presentation. The VR headset has a diagonal field of view (FOV) of 96°. The experiment was designed in Unity 4.6.7f1. The tracking camera of the Oculus Rift was mounted on a separate stand positioned on the table. The participant held a Microsoft Xbox 360 controller, moved the pointer with the left hand using a joystick and confirmed the decision by pressing a button with the right hand.

### 2.4. Stimuli and design

**Fig 2:**
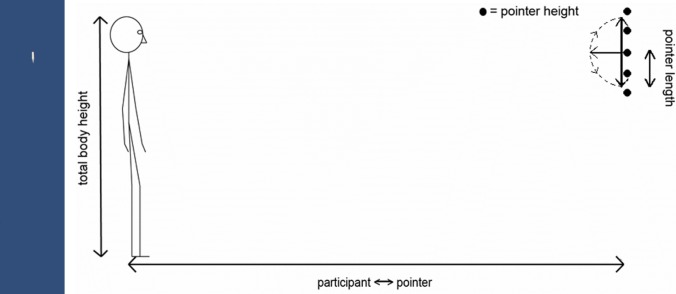
The pointer stimulus and a schematic overview of the setup used in the experiment. (A) An example image of the pointer stimulus, here with an angle of + 48.2° from straight down, showing a field of view of about 20° horizontal and 100° vertical (out of about 100° total for both directions) (left image). (B) The dotted line indicates the range of possible pointer rotations. The pointer heights were chin height and the chin +/- 1/12 and +/- 2/12 of total body height (right image).

The virtual environment consisted of empty space with a blue background. On each trial, the participant saw a round pointing stick with a blunt backside and a pointy front side (see Fig 2A). The backside of the pointer was fixed to a virtual (non-visible) vertical plane orthogonal to the participant’s viewing direction at 1.3 m distance from the participant (the distance of vergence in this VR headset). The pointer had a virtual length of 25 cm and a diameter of 2 cm, was a light-grey colour and had a fixed lighting source straight above.

The starting direction of the pointer was pointing straight down or straight up, at one of five fixed backside heights: the participant’s chin height, chin height +/- 1/12 of the participant’s total body height, chin height +/- 2/12 of body height (see schematic in Fig 2B). As in the Alsmith and Longo [6] study, the independent variables, pointer starting direction and pointer height, were included to test for their possible influences on participants’ judgements, as well as to make the task more diverse. Ten blocks of trials were administered, each containing one trial of every type in random order, making a total of one hundred trials.

The experiment had a within-subject design with three factors: 2 x pointer starting direction, 5 x pointer height and 10 x repetition (blocks), and one measure: scored body region (data analysis was done in terms of percentages of trials per body region; for the computation of the measure see section 2.6)

### 2.5. Task

The participants received the following instructions: in English: "Your task is to adjust the direction in which the stick is pointing so that it is pointing directly at you", or in German (the experiment was run completely in German with German speaking participants): "Ihre Aufgabe ist es, die Richtung des Zeigestocks so zu verändern, dass dieser genau auf Sie zeigt". To perform the task, the participant used the joystick on the left-hand side of a controller to rotate the pointer upwards or downwards (both directions were permitted at all times) through their sagittal plane, with the speed proportional to the pressure administered on the joystick (maximum speed was 75°/s). They confirmed their preferred position by pressing a button on the right-hand side of the controller. Participants were asked to respond as accurately and quickly as possible and to stand still throughout the experiment.

### 2.6. Analysis

The measure recorded during the experiment was the angle of the pointer with the virtual plane to which its backside was fixed (with a range from 0° for completely down and 180° for completely up), when the participant indicated that the pointer was pointing “directly at you”. Using the individualised height of the pointer, this angle was recomputed into the height at which the virtual extension of the pointer would intersect with the plane of the participant’s body. As in Alsmith and Longo [6], depending on this intersection with the body each response was coded as falling into one of seven bodily regions, based on individual body measurements: below the torso (= below the hips), lower torso (= between the hips and the elbows), upper torso (= between the elbows and the shoulders), neck (= between the shoulders and the chin), lower face (= between the chin and the nose), upper face (= between the nose and the top of the head (= total body height)), and above the head (= above total body height; this region is added for classification, because we found a substantial amount of pointing here). These regions were chosen according to visually salient boundaries to facilitate coding, which correspond roughly to nameable body parts.

The numbers of responses for the different body regions, were analysed using a repeated measures analysis of variance (RM-ANOVA), with within-subject factors pointer starting direction (2 levels), pointer height (5 levels) and body region (5 levels), and ᾱ = .05.

It was tested whether a significant correlation was present between the pointing heights in the VR setup and on the body template.

### 2.7. Results

#### VR pointing task

**Fig 3:**
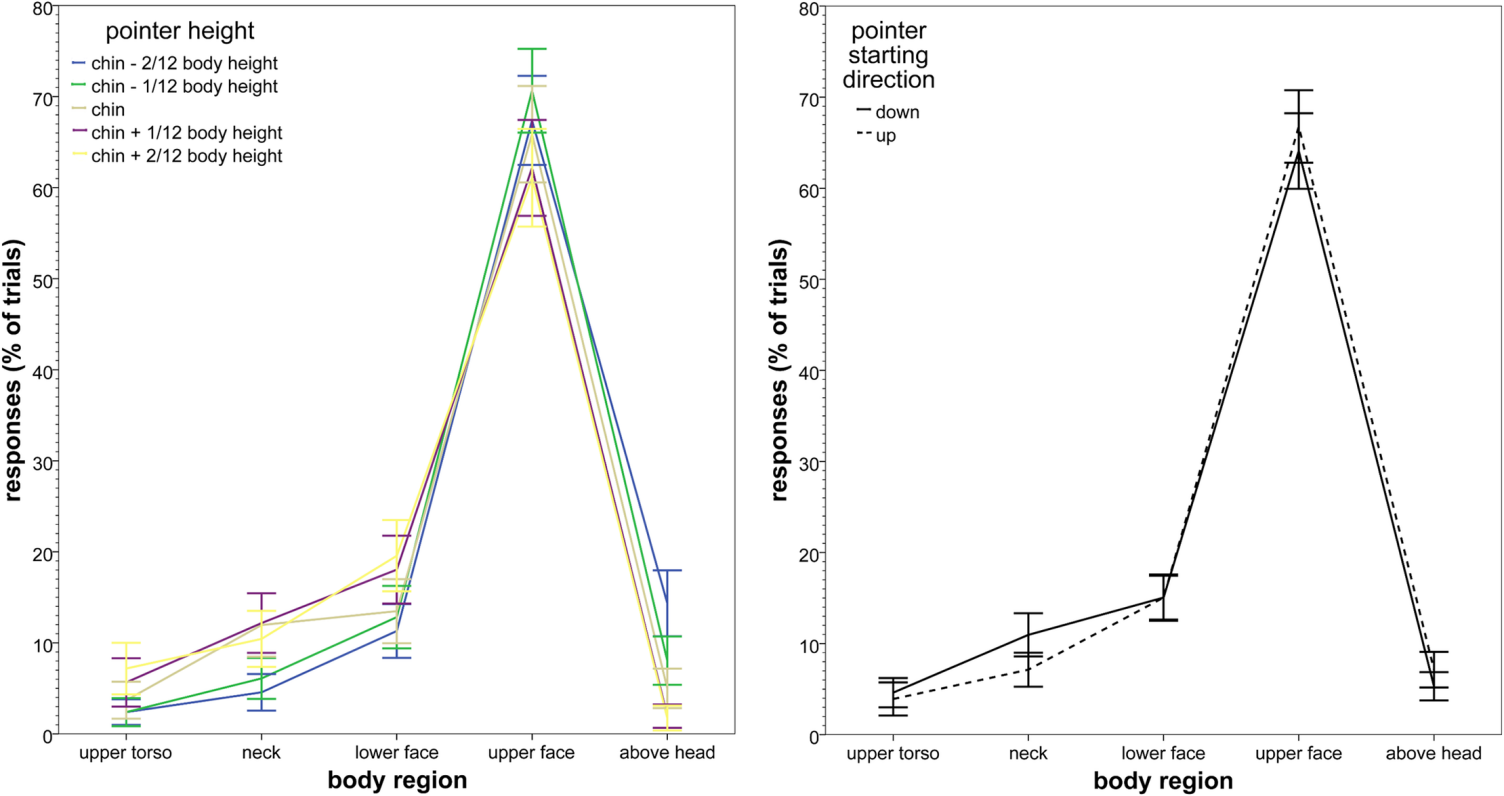
Body region pointed at for pointing at self. A (left). By pointer height, in percentage of trials (error bars: +/- 2 SE). B (right). By pointer starting direction, in percentage of trials (error bars: +/- 2 SE).

None of the responses were scored as below the upper torso. Therefore, no body regions below the upper torso were included in further analyses. All results reported here are Greenhouse-Geisser corrected, because of failed Mauchly’s tests of sphericity. There was a significant main effect of body region (*F*(1.58, 34.8) = 59.2, *p* < .001, η_p_^2^ = .73), indicating that the responses were not evenly distributed across the different body regions. Overall, a strong preference can be seen for pointing at upper face. This effect of region was modulated by significant interactions between body region and pointer height (*F*(5.40, 119) = 4.43, *p* = .001, η_p_^2^ = .17; see Fig 3A). There was no significant interaction between body region and pointer starting direction (*F*(2.99, 65.8) = 2.24, *p* = .092, η_p_^2^ = .092; see Fig 3B), nor a three-way interaction between body region, pointer starting position and pointer height (*F*(5.19, 114) = .58, *p* = .723, η_p_^2^ = .026).

The interaction between body region and pointer height showed the following pattern: a larger proportion of the pointing at higher regions (upper face and above head) for the lower pointer heights (chin– 2/12 and chin– 1/12 of total body height) and a larger proportion of the pointing at lower regions (upper torso, neck and lower face) for the higher pointer heights (chin height, chin + 1/12 and chin + 2/12 of total body height) (with, in absolute numbers, the upper face being the most prevalent for each pointer height).

#### Body template task

**Fig 4:**
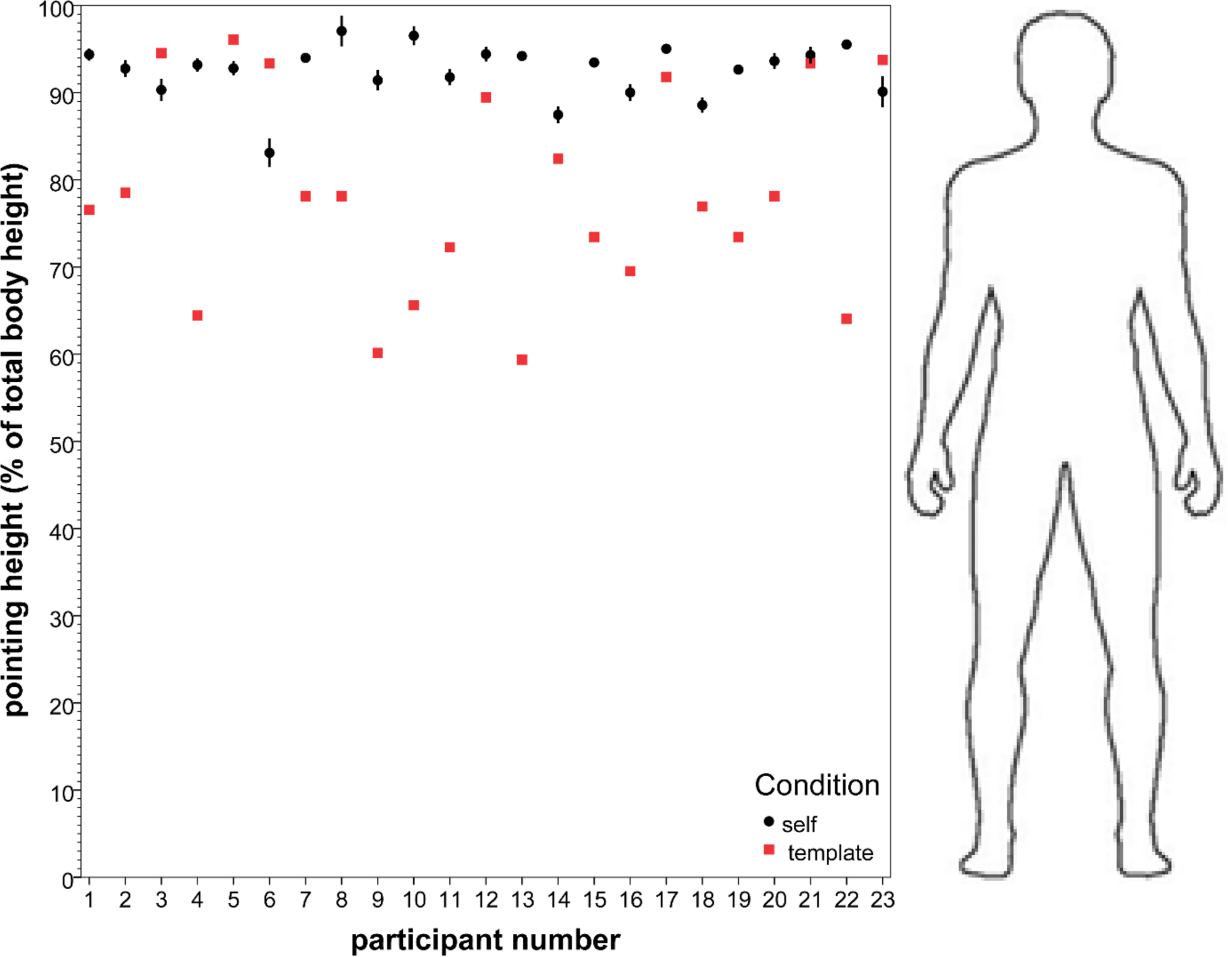
Pointing at self on one's own body compared to on the body template. The mean pointing height on the body for the VR pointing task (in percentage of total physical body height) compared to on the body template task (in percentage of total template body height), per participant (error bars: +/- 2 SE). An image of the body template is added to provide reference, in terms of the template used, as well as in terms of an approximately average body.

Pointing on the body templates was found to be lower on the body (*M* = 78.4 (% of total template body height), *SD* = 11.7, n = 23) than in the VR setups (*M* = 92.5 (% of total physical body height), *SD* = 3.2, n = 23) on a paired-samples t-test (*t*(22) = 5.17, *p* < .001, Cohen's d_z_ = 1.09) (see Fig 4). More than half of the participants pointed to the upper torso, and the rest to the (upper) face in the body template task, while pointing to self in the VR setup was primarily to the upper face.

No significant correlation was present between the pointing to self in the VR setup and on the body template (*r* = -.297, n = 23, *p* (two-tailed) = .169).

#### Survey questions

On the survey question "Did you use a specific strategy for deciding where to direct the pointer? yes/no If so, what did you do?" it was reported by the large majority of participants that they had tried to point to the head or the eyes (16 out of the total of 23, vs 2 with the upper torso or the heart as chosen target and 5 with "no strategy"), either directly (4 head and 8 eyes), or indirectly (4) by making the pointer appear like a dot.

## 3. Discussion

The main finding in the present experiment was the overall strong preference for pointing at the upper face when asked to “point directly at you” while wearing a VR Headset. As in Alsmith and Longo's [6] study, the number of responses towards the upper torso and the neck—possibly interesting regions with regard to embodied self-location—were not extremely small though (4.3 and 9.0 percentage of the total number of responses, respectively). Alsmith and Longo found a very small number of responses towards the lower torso, in the present study there were none.

Employing the experimental design from the Alsmith and Longo [6] study, their bimodal result of upper torso and upper face as main locations where participants on average indicated themselves to be was thus not found to occur with a VR headset. Also, their finding that participants stopped at the overall preferred regions which they reached first, was not replicated in the current study: only one overall preferred regions was found and, moreover, no significant interaction between body region and pointer starting direction was present.

The predominant pointing to the upper face found here, may show the spread of pointing across one area considered to be the location of the self, i.e. the upper part of the face or head, or it may result from (inaccuracy in) pointing to one specific location within this larger area, e.g. the eyes. However, the specific pattern found seems not to be the result of averaging over participants, as most individual participants (19 out of the 23) showed a clear preference for pointing at the upper face.

The pointing found here being largely to the upper face may (partially) be a result of the technical setup used. Wearing the VR headset, participants did not have any visual access to their bodies, possibly reducing their ability to point to other parts of their body than the face (and neck), to which normally they would have visual access. Moreover, this lack of visual access to their body may also have promoted participants to point at the experienced origin of their perception, which is typically the eyes (or a derived egocentre located in the head).

Egocentric distance (the distance from oneself to another location) has typically been found to be underestimated in VR headsets [17,18] and may have played a role in our results. However, egocentric distance has been found to be less underestimated in the Oculus Rift, than in older VR headsets [19–20], although results are still somewhat mixed and seem to partially depend on the measure of distance estimation used [21].

Additionally, the (upper) face may have been highly salient and the area of the body most easily located, as a result of sensations of weight and pressure from wearing the headset. To further test for effects of visual access to one's own body, potential VR headset related visual distortions, as well as of having a heavy piece of equipment on one's head, in follow-up studies different virtual reality setups should be employed, including one using a large immersive screen and not a headset. Additionally, in future studies this methodology should be used with richer cue environments.

Visual access to one's own body was also not provided by giving the participant a self-avatar in the virtual environment or by instead using augmented reality. Another potentially interesting future study would therefore be to investigate whether a self-avatar would result in different self-pointing behaviour.

In several clinical conditions distortions of body representations are involved. Recently, also in healthy participants structural distortions of body representations have been found [22–24]. Possibly these play some role in either or both of our tasks.

In the body template task participants most often indicated the upper torso as where they were located, followed by the (upper) face. This difference with the findings for the pointing to self in the current 1PP experiments in VR (largely to the upper face) is not in line with the studies discussed earlier [4,5,7,8], which all reported (locations related to) the face as the self-location found most. An important distinction between the VR task and the body template task was that participants had visual access to their own body in the body template task and were not wearing the VR headset anymore, which might be reasons for the different results between the two tasks. A possible way to evaluate this would be to do the body template task or a similar task also in the VR headset.

Starmans and Bloom [7,8] found very similar results for self-location in children and in adults (mainly the head, more particularly in or near the eyes). This combined with their specific tasks getting at self indirectly (judging when objects are closer to a person and erasing as much of a picture of a person while leaving the person in), they interpreted as support for the idea that experienced self-location is not so much based on cultural learning but rather has a natural-intuitive character. Answers on our survey questions showed the participants in the current study to come from a diversity of cultural backgrounds (with regard to being religious or not, nationality and countries lived in). No systematic comparison for self-location between cultural backgrounds was included in this study though. Considering that the notion of self-location under study here could well be (in part) socially or culturally constructed, a controlled follow-up study across cultures would be of interest.

The large majority of participants reporting in the survey that they had tried to point to the head or the eyes in the VR task, indicates that it had been a conscious strategy for most participants to point to a region or location in the upper face. Regardless what the underlying cause(s) for the strong behavioural preference for the upper face may have been, the intended strategies were thus largely in line with it.

These results suggest that wearing a VR headset might alter where people locate themselves, specifically making them more head-centred. More research is needed to determine if this is true for different virtual reality technologies such as augmented displays or large screen immersive displays, as well as in richer cue environments including (self-)avatars.

## Supporting information

S1 Questionnaire The set of survey questions the participants answered on paper at the end of the experimental session. (PDF)

S1 Dataset The complete merged raw dataset, with variable annotations. (XLSX)
