# Supplementary material for: Where am I in virtual reality?
Source: PLoS One. 2018 Oct 10;13(10):e0204358. doi: 10.1371/journal.pone.0204358 (PMC6179224; doi:10.1371/journal.pone.0204358)
Supplement: S1 Questionnaire — (PDF) [file pone.0204358.s001.pdf]

Post-questionnaire

Please circle the relevant answers, give number ratings where asked, and fill out the open questions.

Participant nr.: ____

Date: ____

Time: ____

1. Did you understand the tasks? yes no
2. Were the tasks clear and easy to understand? yes no

If not, what was unclear or difficult? ____________________________________

____

____

3. Did you use a specific strategy for deciding where to direct the pointer to? yes no

If so, what did you do? ____

____

____

4. Did you feel some tasks within one (or more) part(s) of the study were more difficult than others in the same part? yes no

If so, which were more difficult? ____

____

____

5. Do you have any ideas on what the research questions may be? ____________________

   ____

   ____

   ____

6. Do you have anything else on your mind related to the experiment? _______________

   ____

   ____

   ____

7. What is your age? ___________________
8. What is your gender? ________________
9. What is your handedness? right left
10. Do you play sport? yes no

If so, how many hours per week on average? ________________

11. Do you do yoga, Pilates, or something similar? yes no

If so, how many hours per week on average? ________________

12. Do you meditate? yes no

If so, how many hours per week on average? ________________

13. What is your profession/occupation?

14. What percentage of your waking hours do you on average spend

- seated: ____________________
- standing: ___________________
- walking: ____________________
- doing physical labour: _______________

15. At what time did you eat your last meal? ____________________

16. How many hours ago did you eat your last meal?

17. Are you religious? yes no

If so, how many hours per week do you perform specific religious practice on average? ________

18. What is your nationality? ___________________

19. In which country(-ies) did you grow up? ____________________

20. What is your relationship status? ___________________

21. Do you have children? yes no

22. What is the highest level of education you have finished?

23. Do you currently have any pain? yes no

If so, where are you experiencing pain? _____________________

24. Have you experienced virtual reality before? yes no

If so, how many hours in total? ______________

25. Do you play video games? yes no

If so, how many hours per week on average? ________________

26. Did you experience any unpleasant feelings during the experience? yes no

If so, what were they? _______________

27. What do you think was the purpose of the experiment? ____________________________

____

____

1. Please rate your self-confidence (1 (lowest) – 100 (highest)): _________________
2. Please rate your overall stress level (1-100): _________________
3. Please rate your overall happiness (1-100): __________________
4. Please rate yourself on an introversion-extraversion scale (1 (most introvert) – 100 (most extravert)): ___________________
5. Please rate how healthy you currently feel (1-100): __________________
6. Please rate how rested you currently feel (1-100): ___________________
7. How many hours have you slept last night? __________________
8. Please rate how energetic you currently feel (1-100): ________________
9. Please rate how much you enjoyed the experiments (1-100): _________________
10. To what extent did you feel you were doing your best (1-100)? _______________
11. To what extent did the experiments hold your attention (1-100)? ____________________
12. To what extent were you focused on the experiments (1-100)? _______________
13. To what extent were you aware of yourself in your environment (1-100)? _____________
14. To what extent were you aware of your body (1-100)? _______________
15. To what extent did you want to stop the experiment (1-100)? ________________
16. To what extent did you feel separated from your real-world environment (1-100)? _____
